# Tooth loss is associated with an increased risk of hypertension: A nationwide population-based cohort study

**DOI:** 10.1371/journal.pone.0253257

**Published:** 2021-06-15

**Authors:** Ho Geol Woo, Yoonkyung Chang, Ji Sung Lee, Tae-Jin Song

**Affiliations:** 1 Department of Neurology, Kyung Hee University College of Medicine, Seoul, Korea; 2 Department of Neurology, Mokdong Hospital, Ewha Womans University College of Medicine, Seoul, Korea; 3 Clinical Research Center, Asan Medical Center, Seoul, Korea; 4 Department of Neurology, Seoul Hospital, Ewha Womans University College of Medicine, Seoul, Korea; Melaka Manipal Medical College, MALAYSIA

## Abstract

Tooth loss is closely associated with suboptimal oral care. Suboptimal oral care can facilitate local infections. These can lead to systemic inflammation and endothelial dysfunction, which are important pathological mechanisms of hypertension. The aim of this study was to investigate the link between tooth loss and the risk of hypertension. From the national health insurance system-health screening cohort in Korea, 19,680 participants who underwent three or more health examinations, including blood pressure measurements, between January 2003 and December 2008, without any history or diagnosis of hypertension were included in this study. Hypertension was defined as the diagnosis of hypertension (International Classification of Diseases-10 code “I10–11”) accompanied by the prescription of an antihypertensive agent or at least one health examination result of blood pressure of ≥140/90 mmHg. Kaplan-Meier survival curves with the log-rank test were used to evaluate the relationship between oral hygiene indicators and the incidence of hypertension. Cox proportional hazard models were applied to determine the association between oral hygiene indicators and the development of hypertension. During a median follow-up of 7.4 years, 1,853 patients developed hypertension. The estimated incidence of hypertension within seven years was 8.8%. Multivariable analysis confirmed a significant relationship between the number of lost teeth and hypertension (hazard ratio: 2.26; 95% confidence interval [1.24–4.10], p = 0.007, p for trend = 0.005). There was a positive association between the number of lost teeth and the risk of hypertension in a longitudinal research. In conclusion, the number of lost teeth may be associated with the risk of development of hypertension.

## Introduction

Hypertension is an important global health problem, which affected 1.4 billion people in 2010. The number of people with hypertension is expected to substantially exceed 1.6 billion in 2025 [1]. Because hypertension is a major risk factor for morbidity and mortality associated with cardiovascular disease, its treatment and prevention are important [2, 3]. We already knew that hypertension could be prevented by controlling multiple environmental factors such as weight gain, unhealthy diet, excessive dietary sodium, inadequate potassium intake, insufficient physical activity, and alcohol consumption. However, we need further studies on factors associated with the development of hypertension [4].

Tooth loss is a common medical condition in the general population. Inflammation of the gingiva and the gradual destruction of periodontal tissues lead to periodontal disease, which is one of the major causes of tooth loss in adults [5]. On the other hand, good oral hygiene, including frequent tooth brushing and visiting the dentist on a regular basis, is essential to preventing tooth loss [6]. A previous study showed that approximately 30% of patients with tooth loss had periodontitis, and 50% of those with tooth loss had dental caries [7]. In addition, 20% of patients with chronic periodontitis had tooth loss, and 62% of patients with tooth loss had periodontal disease [7, 8]. Moreover, tooth loss can be associated with inflammatory conditions of the gingival, alveolar, and periodontal tissues [9]. Suboptimal oral care can facilitate local infections. These can lead to systemic inflammation and endothelial dysfunction, which are important pathological mechanisms underlying hypertension [10]. In addition, tooth loss may lead to changes in dietary patterns, which can be related to hypertension [11]. A high number of lost teeth was associated with an increased risk of heart failure and the development of cardiovascular diseases [12–14]. However, few studies have reported associations between oral hygiene and the development of hypertension in a longitudinal setting. Thus, the objective of the current study was to investigate a possible association between tooth loss and the risk of hypertension.

## Materials and methods

### Participants

The National Health Insurance System (NHIS) is Korea’s national health insurance system, and it collates various data, such as demographics, income levels, and methods of diagnosis and treatment. The NHIS provides researchers with a random sampling database of health screening and medical information for 50 million Koreans from 2002 to 2015 [15]. The NHIS is operated by the Korean government. The NHIS dataset was updated by annually obtaining health claims data. NHIS members are encouraged to undergo a medical check-up biennially [16]. We recruited and registered the study participants from the NHIS-National Health Screening Cohort (NHIS-HEALS) [15]. Our study used the cohort dataset from the year 2008, as an index year. This cohort includes participants who have undergone medical health screening programs to obtain information about their medical histories, age, sex, income levels, body mass index, comorbidities, health behaviors such as alcohol consumption, smoking status, and regular exercise, blood pressure, laboratory information, and oral hygiene indicators. Oral hygiene and oral health screening were provided to members aged 40 years and older. Self-report questionnaires and dental status examinations by dentists were used for the screening [14].

**Fig 1:**
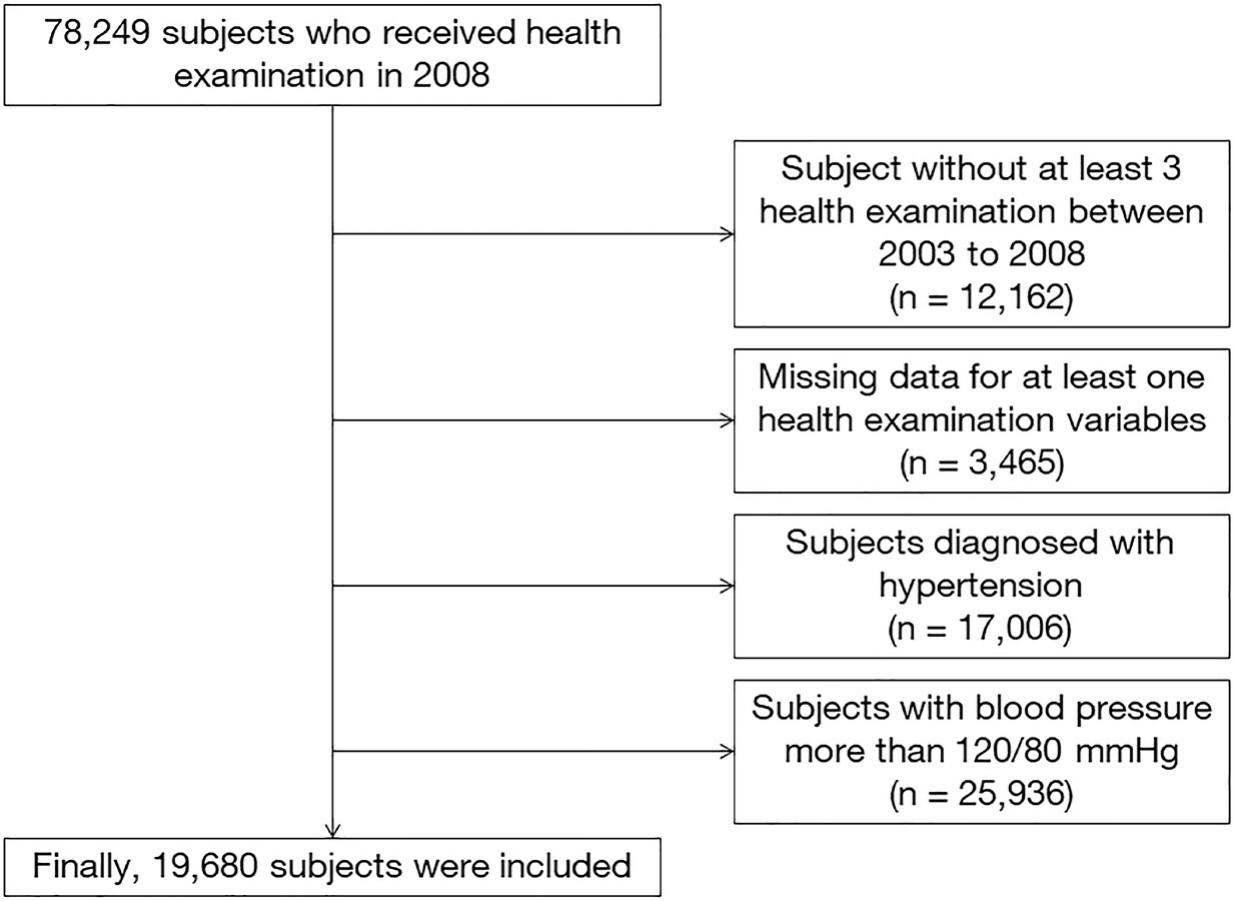
Flowchart showing the selection of study participants.

Among 78,249 participants who received health examinations in 2008, we included 66,087 participants who underwent three or more health examinations between January 1, 2003, and December 31, 2008. We excluded participants with (1) missing data for at least one variable (n = 3,465), (2) history of hypertension, which was defined as the International Statistical Classification of Diseases Related Health Problems (ICD–10) codes ‘I10–11’ and the prescriptions of antihypertensive medications in at least one claim per year (n = 17,006), and (3) systolic blood pressure (SBP) greater than 120 mmHg or diastolic blood pressure (DBP) greater than 80 mmHg detected during any health examination to exclude participants with prehypertension as well (n = 25,936). Finally, 19,680 participants were included in the present study (Fig 1) [14]. Due to the retrospective nature and analysis with the fully anonymized data from NHIS-HEALS, this study was approved by the Institutional Review Board of our institution (Seoul Ewha University Medical Center 2020-08-018). Informed consent was waived, and all investigation was performed according to the Declaration of Helsinki 2013.

### Study variables and definitions

The definition of comorbidity is described in the Supplementary material in a previous study (S1 Appendix). The presence of periodontal disease, including acute and chronic periodontitis, periodontitis, unspecified periodontal disease, and other periodontal diseases, was defined using ICD–10 codes and prescriptions for more than two times by a dentist based on previous studies (K052–056) [14, 17, 18]. Oral hygiene behavior (frequency of tooth brushing, dental visits for any reason, and professional scaling) was obtained from self-reports [14]. Oral hygiene behavior was categorized by the frequency of tooth brushing: 0–1 time, 2 times, and thrice or more per day. Dental visits for any reason and professional scaling were dichotomized as never or at least once per year. The number of lost teeth was ascertained by a dentist during the oral health examinations [14]. We regarded the presence of a fixed dental prosthesis, implant with an abutment, and a third molar as toothlessness. The number of lost teeth was classified into quartiles of 0, 1–7, 8–14, and ≥15, regardless of the cause.

According to guidelines for the management of hypertension, the definition of optimal blood pressure was SBP values of <120 mmHg and/or DBP values of <80 mmHg [19, 20]. Newly developed hypertension was designated as a primary or secondary diagnosis of hypertension (ICD–10 codes I10–I11). The definition of hypertension was based on prescription records of any antihypertensive agent for at least one claim per year and the record of visiting an outpatient clinic or admission. Alternatively, at least one result of SBP greater than 140 mmHg or DBP greater than 90 mmHg from the NHIS-HEALS after the index date was indicative of newly developed hypertension.

### Statistical analysis

To test the normality of the data, we used the Shapiro-Wilk normality test for continuous variables and each p-value was greater than 0.05. Independent t-test and the Chi-squared test were used to compare the continuous and categorical variables, respectively. Kaplan-Meier survival curves with the log-rank test were used to assess the association between the incidence of hypertension and oral hygiene indicators. Seven-year event rates (%) were reported. Cox proportional hazard models were used to estimate the association between oral hygiene indicators and the development of hypertension. The hazard ratio (HR) and 95% confidence interval (CI) were investigated. There was no multicollinearity for the risk of the development of hypertension associated with the oral hygiene indicators in the multivariable analysis (variance inflation factors were less than 5.0, S1 Table). Multivariable Cox regression analyses were used to assess the association between each oral hygiene indicator and the development of hypertension after adjusting for age, sex, income levels, smoking status, alcohol consumption, regular exercise, body mass index, diabetes mellitus, dyslipidemia, renal disease, and history of malignancy in model 1; adjusting for the variables of model 1 and fasting blood sugar, liver panel, and proteinuria in model 2; and adjusting for the variables of model 2 and overall oral hygiene indicators (presence of periodontal disease, frequency of tooth brushing, dental visits for any reason, professional scaling, and the number of lost teeth) in model 3. A goodness-of-fit test for Cox’s proportional hazards models proposed based on Martingale’s residuals was conducted. Models for analyzing the associations between the development of hypertension with the frequency of tooth brushing or the number of lost teeth were fitted (S1 and S2 Figs). For evaluating the proportional hazards for the risk of hypertension, there was no violation of the linear assumption for the risk of hypertension except tooth brushing twice per day (S2 and S3 Tables).

To evaluate the trends in the HRs regarding the frequency of tooth brushing and the number of lost teeth, a p-value was estimated. Subgroup analyses were performed for the interaction between the number of lost teeth and the development of hypertension in relation to demographics and comorbidities. The interaction between each subgroup and oral hygiene was analyzed using Cox proportional hazards regression analyses. Statistical analysis was carried out using SAS software (version 9.2, SAS Institute, Cary, NC, USA). A p-value denoted statistical significance when it was less than 0.05.

## Results

The mean age of the participants was 51.8 years. Of these participants, 58.8% were men, 21.2% were current smokers, 9.5% had diabetes mellitus, and 25.8% had dyslipidemia. Approximately 1.0% of participants had more than eight lost teeth. Based on the examination of periodontitis by a dentist, 40.2% of participants had periodontal disease and 48.8% visited the dentist for any reason. Moreover, 58.3% of the participants brushed their teeth thrice or more per day. Approximately 34.2% of the participants received professional scaling at least once per year (Table 1).

**Table 1:** Baseline characteristics of the study population.

Characteristics	Total
Number of participants	19,680
Age (years)	51.8 ± 5.4
Male sex	11,568 (58.8)
Income levels	
Fifth quintile (highest)	9,838 (50.0)
Fourth quintile	3,536 (18.0)
Third quintile	2,382 (12.1)
Second quintile	2,140 (10.9)
First quintile (lowest)	1,775 (9.0)
Covered by medical aid	9 (0.0004)
Smoking status	
None	13,437 (68.2)
Former smoker	2,080 (10.6)
Current smoker	4,163 (21.2)
Alcohol consumption	8,921 (45.3)
Regular exercise	1,520 (7.7)
Anthropometric measurements	
Body mass index (kg/m^2^)	22.9 ± 2.5
Systolic blood pressure (mmHg)	112.1 ± 5.6
Diastolic blood pressure (mmHg)	71.0 ± 4.7
Comorbidities	
Diabetes mellitus	1,860 (9.5)
Dyslipidemia	5,077 (25.8)
Renal disease	33 (0.2)
History of malignancy	4,384 (22.3)
Medications	
Any antihypertensive agents	677 (3.4)
Any lipid-lowering agents	1,477 (7.5)
Laboratory findings	
Total cholesterol (mg/dL)	195.2 ± 34.2
Fasting blood glucose level (mg/dL)	93.3 ± 18.0
Aspartate aminotransferase (U/L)	24.3 ± 15.6
Alanine aminotransferase (U/L)	22.9 ± 20.5
Gamma-glutamyl transferase (U/L)	29.7 ± 32.2
Proteinuria (≥1+ in dipstick test)	473 (2.4)
Oral health status	
Presence of periodontal disease	7,903 (40.2)
Number of lost teeth	
0	15,424 (78.4)
1–7	4,051 (20.6)
8–14	146 (0.7)
≥15	59 (0.3)
Oral hygiene care	
Dental visits for any reason	9,612 (48.8)
Frequency of tooth brushing (times/day)	
0–1	1,749 (8.9)
2	6,452 (32.8)
≥3	11,479 (58.3)
Professional scaling	6,730 (34.2)

P-value determined using Student’s t-test and Chi-squared test. Data are expressed as the mean ± standard deviation or n (%).

**Fig 2:**
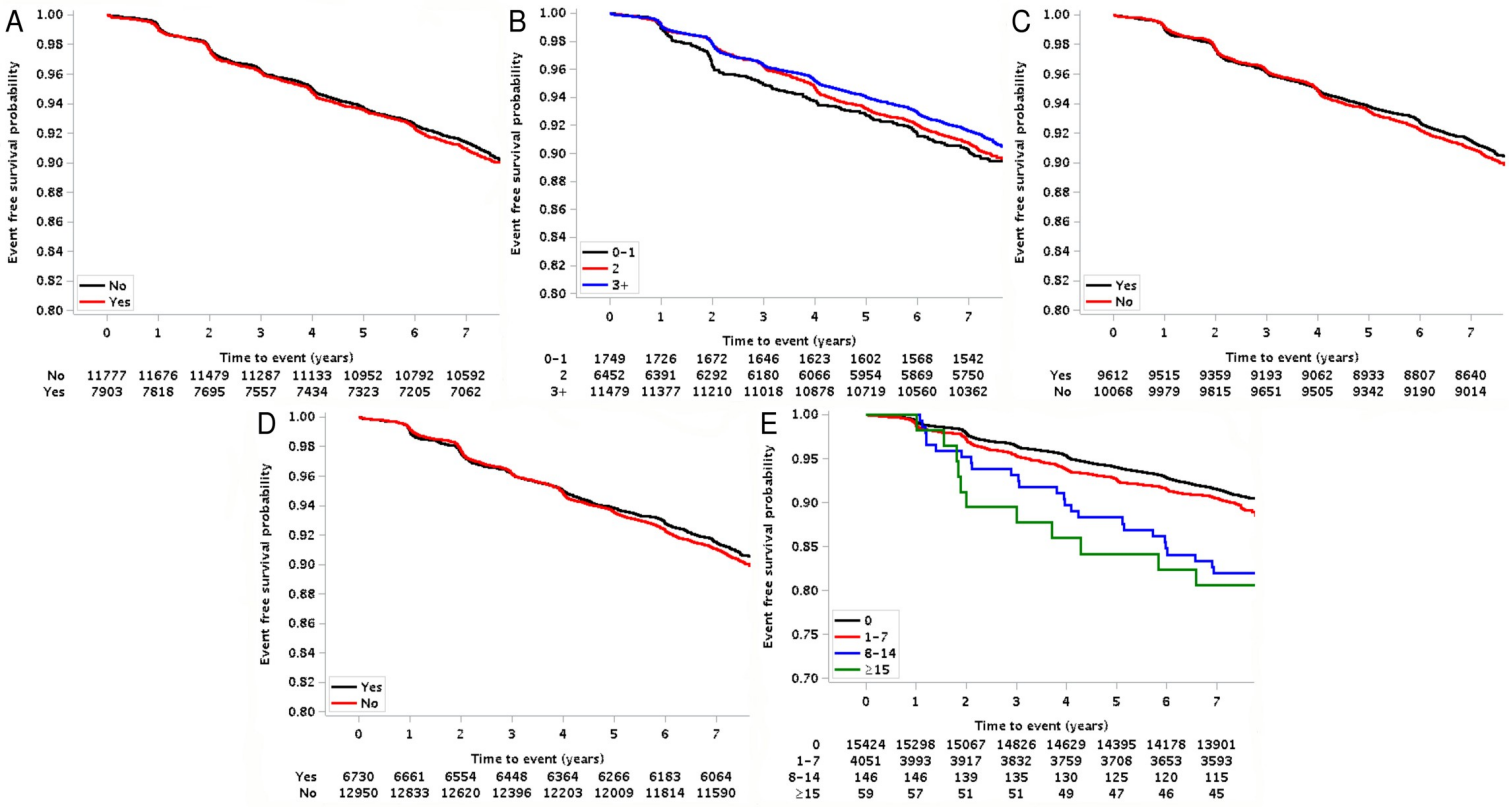
The cumulative incidence curves for newly developed hypertension. Cumulative incidence curves for newly developed hypertension are presented. (A) Presence of periodontal disease, (B) frequency of tooth brushing, (C) dental visits for any reason, (D) professional scaling, and (E) the number of lost teeth.

With a median follow-up of 7.4 years (interquartile range, 7.2–7.6 years), 1,853 cases were diagnosed with newly developed hypertension. The 7-year event rates were 8.8% for newly developed hypertension. Kaplan–Meier survival curves, based on the oral hygiene indicators, for newly developed hypertension are presented in Fig 2. Frequent tooth brushing was negatively associated (p = 0.030) with the risk of developing hypertension while a greater number of lost teeth was positively associated (p < 0.001) with the risk of newly developed hypertension.

The number of lost teeth within the ranges of 8–14 and ≥15 was positively associated with the development of hypertension in the multivariable analysis (HR: 1.97; 95% CI: 1.33–2.92, p < 0.001, p for trend for HR = 0.005 and HR: 2.26; 95% CI: 1.24–4.10, p = 0.007, p for trend for HR = 0.005, respectively) (Table 2). The presence of periodontal disease and variables of oral hygiene did not show significant associations with the development of hypertension in the multivariable analysis, although tooth brushing thrice or more per day was marginally associated with the development of hypertension in the univariable analysis (Table 2).

**Table 2:** Risks of developing hypertension associated with oral hygiene indicators.

	Event rate	Unadjusted model		Multivariable adjusted (1)		Multivariable adjusted (2)		Multivariable adjusted (3)		
	(%), (95% CI)	HR (95% CI)	p value	HR (95% CI)	p value	HR (95% CI)	p value	HR (95% CI)	p value	
Presence of periodontal disease										
No	8.63 (8.12, 9.14)	1 (ref)		1 (ref)		1 (ref)		1 (ref)		
Yes	9.06 (8.42, 9.69)	1.04 (0.95–1.14)	0.412	1.00 (0.91–1.09)	0.924	1.00 (0.91–1.09)	0.943	1.00 (0.91–1.11)	0.947	
Frequency of tooth brushing (times/day)									
0–1	9.75 (8.35, 11.15)	1 (ref)		1 (ref)		1 (ref)		1 (ref)		
2	9.27 (8.56, 9.98)	0.96 (0.81–1.13)	0.609	1.00 (0.85–1.18)	0.995	1.00 (0.85–1.18)	0.995	1.01 (0.85–1.19)	0.918	
≥3	8.39 (7.89, 8.90)	0.86 (0.73–1.00)	0.055	0.98 (0.83–1.15)	0.758	0.98 (0.83–1.15)	0.761	0.99 (0.84–1.17)	0.927	
p for trend		0.010		0.638		0.642		0.821		
Dental visits for any reason										
No	9.07 (8.50, 9.63)	1 (ref)		1 (ref)		1 (ref)		1 (ref)		
Yes	8.52 (7.96, 9.08)	0.94 (0.86–1.03)	0.209	0.94 (0.86–1.03)	0.220	0.94 (0.86–1.03)	0.205	0.95 (0.85–1.06)	0.338	
Professional scaling										
No	8.97 (8.47, 9.46)	1 (ref)		1 (ref)		1 (ref)		1 (ref)		
Yes	8.48 (7.81, 9.15)	0.94 (0.86–1.04)	0.235	0.95 (0.86–1.05)	0.309	0.95 (0.86–1.05)	0.316	0.99 (0.88–1.11)	0.830	
Number of lost teeth										
0	8.49 (8.05, 8.93)	1 (ref)		1 (ref)		1 (ref)		1 (ref)		
1–7	9.50 (8.60, 10.41)	1.15 (1.03–1.28)	0.011	1.06 (0.95–1.19)	0.288	1.06 (0.95–1.18)	0.301	1.06 (0.95–1.19)	0.294	
8–14	18.07 (11.78, 24.36)	2.09 (1.42–3.09)	<0.001	1.93 (1.31–2.86)	0.001	1.97 (1.33–2.91)	<0.001	1.97 (1.33–2.92)	<0.001	
≥15	19.41 (9.11, 29.71)	2.30 (1.27–4.16)	0.005	2.27 (1.25–4.12)	0.007	2.28 (1.25–4.14)	0.007	2.26 (1.24–4.10)	0.007	
p for trend		<0.001		0.005		0.005		0.005		

Event rates are reported as 7-year event rates (%).

Multivariable model (1) was used to evaluate the association of each oral hygiene indicator with the development of hypertension with adjustment for age, sex, income levels, regular exercise, alcohol consumption, smoking status, body mass index (kg/m^2^), diabetes mellitus, dyslipidemia, renal disease, and history of malignancy

Multivariable model (2) was used to evaluate the association of each oral hygiene indicator with the development of hypertension with adjustment for the variables of model 1, as well as systolic blood pressure, fasting blood glucose level, aspartate aminotransferase, alanine aminotransferase, gamma-glutamyl transferase, and proteinuria.

Multivariable model (3) was used to evaluate the association of each oral hygiene indicator with the development of hypertension with adjustment for the variables of model 2, as well as the overall oral hygiene indicators (presence of periodontal disease, frequency of tooth brushing, dental visits for any reason, professional scaling, and number of lost teeth)

CI, confidence interval; HR, hazard ratio.

Trend test for hazard ratios

The subgroup analysis showed no statistically significant interaction between the number of lost teeth and the development of hypertension related to age, sex, alcohol consumption, smoking status, regular exercise, diabetes mellitus, and dyslipidemia (S4 Table).

## Discussion

The key finding of our study is that the number of lost teeth (≥8) may be associated with the risk of development of hypertension. Previous studies have shown that tooth loss is associated with hypertension. Partial tooth loss was related to a higher risk of having hypertension among middle-aged and older adults in India (odds ratio: 1.62) [21]. The association between tooth loss (>10) and hypertension was also observed in patients younger than 65 years in a French cohort (odds ratio: 1.17) [22]. Another cross-sectional study reported that tooth loss (>15) was related to severe hypertension in participants aged ≥50 years in China [23]. A cross-sectional study involving the Korean population showed that a decrease in the number of teeth may be independently associated with hypertension [24]. Our results are similar to those of these previous studies and provide additional information on the positive and dose-dependent association between tooth loss and an increase in the risk of developing hypertension in a longitudinal study including a Korean nationwide randomly sampled population.

In our study, the presence of periodontal disease, dental visits for any reason, and professional scaling did not show any significant association with the development of hypertension in the univariable analysis. In a recent meta-analysis, moderate to severe periodontal disease (odds ratio: 1.22; 95% CI: 1.10–1.35) and severe periodontal disease (odds ratio: 1.49; 95% CI: 1.09–2.05) were associated with hypertension [25]. This discrepancy between our results and the previous meta-analysis may be attributed to the differences in method, participants, the definition for the presence of periodontal disease, and the methods for defining newly developed hypertension [26]. With the increasing frequency of tooth brushing, the prevalence of hypertension decreased even after adjusting for various vascular risk factors and the presence of periodontitis in a previous study [27]. In contrast, in our study, tooth brushing thrice or more per day was marginally related to new-onset hypertension in the univariable analysis. These findings suggest that other confounding variables are more strongly associated with the development of new-onset hypertension than the frequency of tooth brushing.

Our study did not demonstrate the mechanism underlying the association between new-onset hypertension and the number of lost teeth and whether tooth loss was a direct risk factor for the development of hypertension and individuals with different oral diseases or poor oral hygiene were more susceptible to hypertension. However, several hypotheses have been put forward to explain the link. Tooth loss may lead to alterations of dietary patterns, such as low intake of citrus fruit, beta carotene, folate, vitamin C, and fiber. These undesirable eating habits are closely related to the development of hypertension [11, 28, 29]. Our study showed that the loss of 8 or more teeth was associated with hypertension. Having 20 or more teeth (≤ 8 tooth loss) is enough for masticatory function. Tooth loss is associated with a lesser intake of vitamins and fiber and a higher intake of cholesterol, leading to an increased risk of hypertension. Therefore, the association between tooth loss and hypertension may be explained by nutritional intake. In addition, tooth loss and inflammation of periodontal pockets due to removed teeth may lead to chronic systemic inflammation and increase the risk of hypertension [30].

The current study has some limitations. First, the results cannot be generalized to populations of different ethnicities because the data set only included the data of Koreans. Second, the definition of the presence of periodontal disease based on the ICD–10 code found in health claim data does not reflect the recently published case definitions and classification criteria for periodontal disease [26]. Therefore, a further study using the recently updated periodontal disease classification is needed. Third, dental health check-up data in our study did not have information on the cause and time of tooth loss, plaque or periodontal disease indices, and the dental caries index. There were also no data on the severity of periodontitis, baseline pocket depth, and clinical attachment loss/level. Fourth, the dental health check-up data in our study did not have data on whether periodontitis was examined by specialist periodontists or general practitioners. Fifth, because data on oral hygiene were collected from a self-reported survey, there may have been recall bias. Sixth, although this study attempted to investigate the fixed effect of variables, except blood pressure, and used a retrospective observational design, serial dental health data for the period between 2003 and 2008 were not evaluated. Seventh, because a high censoring rate (90.57% in our study) can cause lower accuracy and effectiveness of the results of the analysis, the risk of bias may increase (S5 Table). Finally, the NHIS-HEALS does not contain information, including education levels, marital status and blood inflammatory markers. Despite these limitations, it is necessary to be aware of the risk of developing hypertension and monitor the development of hypertension in patients with tooth loss. In addition, for addressing the limitation, further studies using a validated dataset for periodontitis with indexes for specific periodontal diseases or a dataset with data on periodontal disease confirmed by periodontitis specialists or calibrated dentists are needed.

## Conclusions

Our study demonstrated that the loss of 8 or more teeth was associated with a higher risk of developing hypertension in a longitudinal study setting. Thus, the higher number of lost teeth may be associated with the risk of new-onset hypertension.

## Supporting information

S1 Checklist STROBE statement—checklist of items that should be included in reports of observational studies. (DOC)

S1 Fig Goodness of fit assessments for association with frequency of tooth brushing. (TIF)

S2 Fig Goodness of fit assessments for association with number of tooth loss. (TIF)

S1 Appendix Supplementary material methods. (DOCX)

S1 Table Multicollinearity assessment of the risk of new-onset hypertension according to oral hygiene indicators. (DOCX)

S2 Table Test for non-proportional hazards assumption for risk of hypertension. (DOCX)

S3 Table Test for increasing discrimination power for Cox regression model. (DOCX)

S4 Table The subgroup analysis regarding the number of lost teeth and new-onset hypertension in association with demographics or comorbidities. (DOCX)

S5 Table Summary of the number of event and censored in this study. (DOCX)
