# Supplementary figures and images for: Tooth loss is associated with an increased risk of hypertension: A nationwide population-based cohort study
Source: PLoS One. 2021 Jun 15;16(6):e0253257. doi: 10.1371/journal.pone.0253257 (PMC8205122; doi:10.1371/journal.pone.0253257)

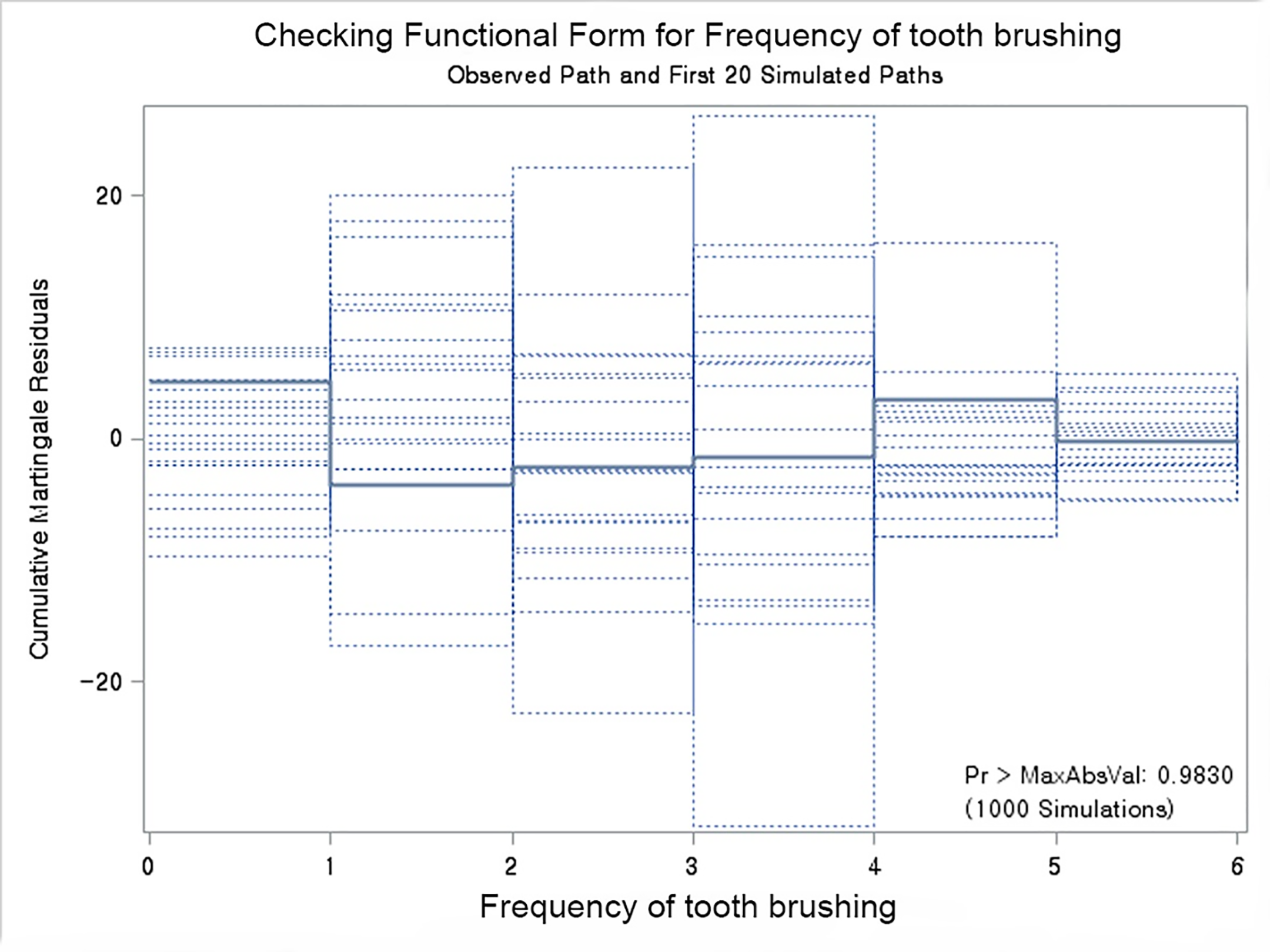

Supplement: S1 Fig — (TIF) [file pone.0253257.s002.tif]

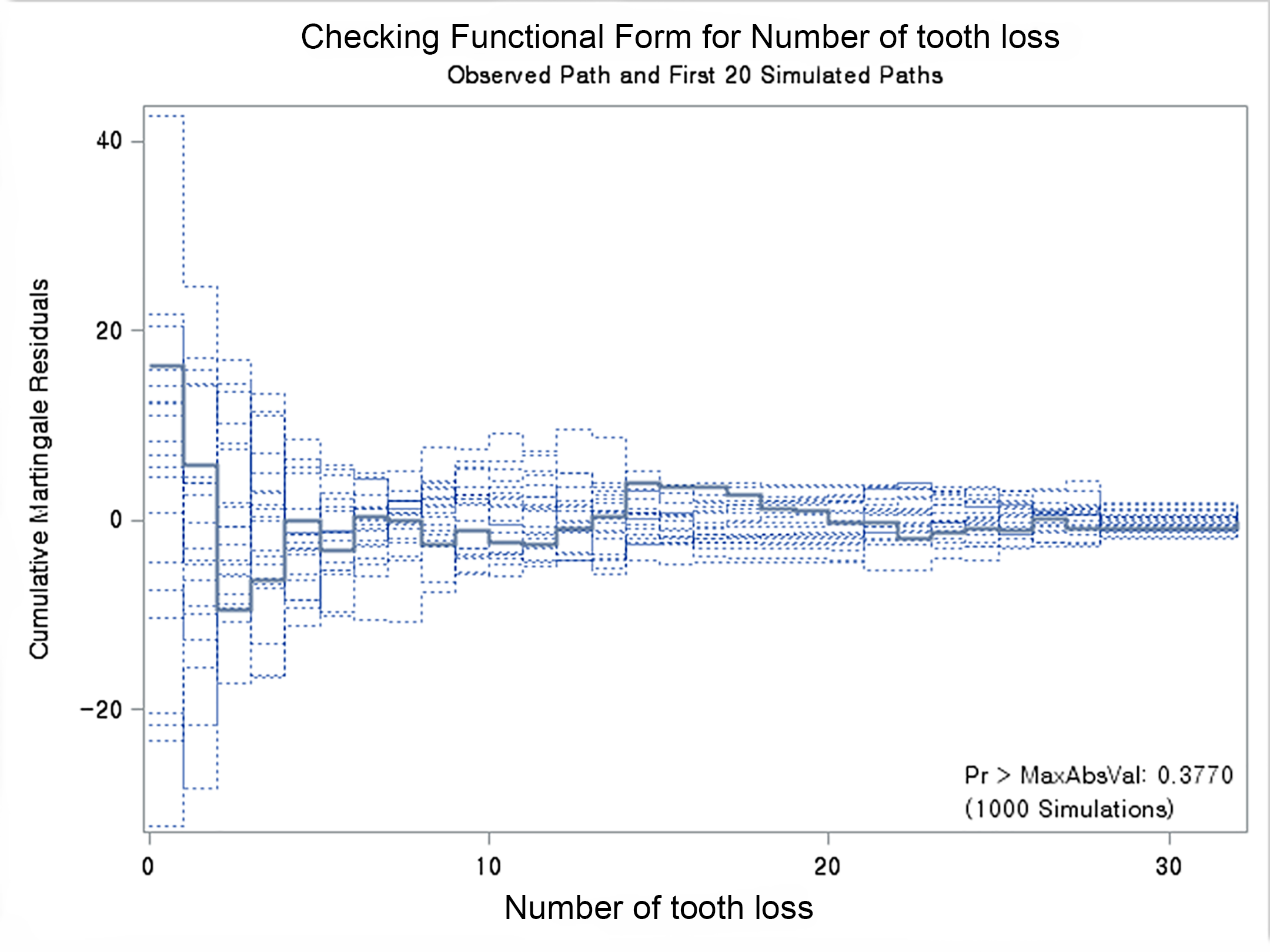

Supplement: S2 Fig — (TIF) [file pone.0253257.s003.tif]
