# Supplementary material for: Tooth loss is associated with an increased risk of hypertension: A nationwide population-based cohort study
Source: PLoS One. 2021 Jun 15;16(6):e0253257. doi: 10.1371/journal.pone.0253257 (PMC8205122; doi:10.1371/journal.pone.0253257)
Supplement: S1 Appendix — (DOCX) [file pone.0253257.s004.docx]

**S1 Appendix. Supplementary material methods**

**The definition of comorbidity**

Information on smoking status and alcohol consumption were obtained by questionnaire. Body mass index was defined as the participant’s weight in kilograms divided by the square of the participant’s height in meters. Regular exercise was considered to be strenuous physical activity performed for at least 20 min more than once per week [1]. Smoking status was categorized into none, former smoker and current smoker regardless of the amount of smoking. Current smoker was defined as a person who had smoked more than 5 packs (100 cigarettes) in a lifetime as defined by the World Health Organization and smoked daily or occasionally for the last 28 days. Former smoker was defined as a person who had smoked more than 100 cigarettes in a lifetime but did not smoke in the last 28 days [2]. Diabetes mellitus was defined as main or secondary diagnosis of diabetes mellitus (International Statistical Classification of Diseases Related Health Problems [ICD–10] code ‘E10–E14’). Criteria for this diagnosis included at least one claim per year for both visiting an outpatient clinic and admission accompanied by prescription records for any hypoglycemic agents. Alternatively, at least one fasting plasma glucose ≥126 mg/dL. Dyslipidemia was defined using ICD–10 code E78 and the prescription of a lipid-lowering agent including statins with at least one claim per year. Renal disease was identified by ICD–10 codes N18.1–N18.5 and N18.9 with at least one claim per year. Malignancy was defined by ICD–10 codes C00–D48 with at least one claim per year. Body mass index and blood and urine laboratory findings were used with the baseline dataset [3].

**References**

1. Kim MK, Han K, Joung HN, Baek KH, Song KH, Kwon HS. Cholesterol levels and development of cardiovascular disease in Koreans with type 2 diabetes mellitus and without pre-existing cardiovascular disease. Cardiovasc Diabetol. 2019;18(1):139. doi: 10.1186/s12933-019-0943-9. PubMed PMID: 31640795; PubMed Central PMCID: PMCPMC6805335.

2. Lee KH, Lee CM, Kwon HT, Oh S-W. Relationship between Obesity and Smoking in Korean Men: Data Analyses from the Third and Fourth Korea National Health and Nutrition Examination Surveys (KNHANES). JKSRNT. 2010;1(2):115-23. Epub 07/15. doi: 10.25055/JKSRNT.2010.1.2.115.

3. Park SY, Kim SH, Kang SH, Yoon CH, Lee HJ, Yun PY, et al. Improved oral hygiene care attenuates the cardiovascular risk of oral health disease: a population-based study from Korea. Eur Heart J. 2019;40(14):1138-45. doi: 10.1093/eurheartj/ehy836. PubMed PMID: 30561631.
