# Supplementary material for: Tooth loss is associated with an increased risk of hypertension: A nationwide population-based cohort study
Source: PLoS One. 2021 Jun 15;16(6):e0253257. doi: 10.1371/journal.pone.0253257 (PMC8205122; doi:10.1371/journal.pone.0253257)
Supplement: S1 Table — (DOCX) [file pone.0253257.s005.docx]

**S1 Table. Multicollinearity assessment of the risk of new-onset hypertension according to oral hygiene indicators**

|  | Variance Inflation Factors |
| --- | --- |
| Age (years) | 1.1379 |
| Male sex | 1.5436 |
| Income levels, % (n) |  |
| Fifth quintile (highest) | - |
| Fourth quintile | 1.2226 |
| Third quintile | 1.2372 |
| Second quintile | 1.2579 |
| First quintile (lowest) | 1.2378 |
| Covered by medical aid | 1.0021 |
| Regular exercise | 1.0154 |
| Alcohol consumption | 1.2651 |
| Body mass index (kg/m2) | 1.0748 |
| Diabetes mellitus | 1.3750 |
| Dyslipidemia | 1.4765 |
| Current smoker | 1.2452 |
| Renal disease | 1.0044 |
| History of malignancy | 1.0194 |
| Total cholesterol | 1.4767 |
| Fasting blood glucose level | 1.3946 |
| Aspartate aminotransferase | 4.9520 |
| Alanine aminotransferase | 4.8928 |
| Gamma-glutamyl transferase | 1.2594 |
| Proteinuria | 1.0065 |
| Presence of periodontal disease | 1.1021 |
| Frequency of tooth brushing (time/day) |  |
| 0-1 | - |
| 2 | 3.0139 |
| ≥3 | 3.1169 |
| Dental visits for any reason | 1.5237 |
| Professional scaling | 1.5021 |
| Number of lost teeth |  |
| 0 | - |
| 1-7 | 1.0499 |
| 8-14 | 1.0254 |
| ≥15 | 1.0198 |
