# Supplementary material for: Tooth loss is associated with an increased risk of hypertension: A nationwide population-based cohort study
Source: PLoS One. 2021 Jun 15;16(6):e0253257. doi: 10.1371/journal.pone.0253257 (PMC8205122; doi:10.1371/journal.pone.0253257)
Supplement: S2 Table — (DOCX) [file pone.0253257.s006.docx]

**S2 Table. Test for non-proportional hazards assumption for risk of hypertension**

| **zph Tests for Nonproportional Hazards** | | | | | | |
| --- | --- | --- | --- | --- | --- | --- |
| **Transform** | **Variable** | **Correlation** | **ChiSquare** | **Pr > ChiSquare** | **t Value** | **Pr > \|t\|** |
| RANK | Periodontal disease | 0.0167 | 0.5191 | 0.4712 | 0.72 | 0.4724 |
| RANK | Frequency of tooth brushing (2 times/day) | 0.0499 | 4.6321 | 0.0314 | 2.15 | 0.0318 |
| RANK | Frequency of tooth brushing (≥3 times/day) | 0.0337 | 2.1452 | 0.1430 | 1.45 | 0.1468 |
| RANK | Dental visits for any reason | -0.0092 | 0.1670 | 0.6828 | -0.39 | 0.6929 |
| RANK | Professional scaling | -0.0219 | 0.9230 | 0.3367 | -0.94 | 0.3456 |
| RANK | Number of tooth loss (1-7) | -0.0451 | 3.8465 | 0.0499 | -1.94 | 0.0521 |
| RANK | Number of tooth loss (8-14) | 0.0065 | 0.0787 | 0.7791 | 0.28 | 0.7790 |
| RANK | Number of tooth loss (≥15) | -0.0257 | 1.2142 | 0.2705 | -1.11 | 0.2692 |
