# Supplementary material for: Tooth loss is associated with an increased risk of hypertension: A nationwide population-based cohort study
Source: PLoS One. 2021 Jun 15;16(6):e0253257. doi: 10.1371/journal.pone.0253257 (PMC8205122; doi:10.1371/journal.pone.0253257)
Supplement: S3 Table — (DOCX) [file pone.0253257.s007.docx]

**S3 Table. Test for increasing discrimination power for Cox regression model**

| **Harrell's Concordance Statistic** | | | | | |
| --- | --- | --- | --- | --- | --- |
| **Source** | **Estimate** | **Comparable Pairs** | | | |
|  |  | **Concordance** | **Discordance** | **Tied in Predictor** | **Tied in Time** |
| Model | 0.6133 | 20751570 | 13083560 | 0 | 1015 |
