# Supplementary material for: Tooth loss is associated with an increased risk of hypertension: A nationwide population-based cohort study
Source: PLoS One. 2021 Jun 15;16(6):e0253257. doi: 10.1371/journal.pone.0253257 (PMC8205122; doi:10.1371/journal.pone.0253257)
Supplement: S4 Table — (DOCX) [file pone.0253257.s008.docx]

**S4 Table. The subgroup analysis regarding the number of lost teeth and new-onset hypertension in association with demographics or comorbidities**

|  | Model 1 | | Model 2 | | Model 3 | |
| --- | --- | --- | --- | --- | --- | --- |
|  | HR (95% CI) | p value for interaction effect | HR (95% CI) | p value for interaction effect | HR (95% CI) | p value for interaction effect |
| Age |  | 0.334 |  | 0.326 |  | 0.322 |
| ≤50 |  |  |  |  |  |  |
| Number of lost teeth |  |  |  |  |  |  |
| 0 | 1 (reference) |  | 1 (reference) |  | 1 (reference) |  |
| 1-7 | 0.77 (0.51–1.17) |  | 0.77 (0.51–1.16) |  | 0.77 (0.51–1.16) |  |
| 8-14 | 3.80 (1.31–10.99) |  | 3.82 (1.32–11.06) |  | 3.77 (1.30–10.92) |  |
| ≥15 | 2.20 (0.14–35.53) |  | 2.19 (0.14–35.27) |  | 2.13 (0.13–34.30) |  |
| ≥51 |  |  |  |  |  |  |
| Number of lost teeth |  |  |  |  |  |  |
| 0 | 1 (reference) |  | 1 (reference) |  | 1 (reference) |  |
| 1-7 | 1.04 (0.90–1.20) |  | 1.03 (0.89–1.19) |  | 1.02 (0.88–1.18) |  |
| 8-14 | 1.97 (1.45–2.66) |  | 1.92 (1.42–2.59) |  | 1.85 (1.37–2.50) |  |
| ≥15 | 3.05 (2.30–4.04) |  | 2.96 (2.24–3.92) |  | 2.82 (2.13–3.74) |  |
| Sex |  | 0.921 |  | 0.942 |  | 0.953 |
| Men |  |  |  |  |  |  |
| Number of lost teeth |  |  |  |  |  |  |
| 0 | 1 (reference) |  | 1 (reference) |  | 1 (reference) |  |
| 1-7 | 0.97 (0.81–1.15) |  | 0.96 (0.81–1.15) |  | 0.97 (0.81–1.15) |  |
| 8-14 | 1.40 (0.94–2.10) |  | 1.40 (0.94–2.10) |  | 1.40 (0.93–2.09) |  |
| ≥15 | 2.49 (1.12–2.55) |  | 2.27(1.09–2.49) |  | 2.31 (1.07–2.46) |  |
| Women |  |  |  |  |  |  |
| Number of lost teeth |  |  |  |  |  |  |
| 0 | 1 (reference) |  | 1 (reference) |  | 1 (reference) |  |
| 1-7 | 1.01 (0.81–1.25) |  | 1.00 (0.81–1.24) |  | 1.00 (0.81–1.24) |  |
| 8-14 | 1.38 (0.90–2.09) |  | 1.36 (0.89–2.06) |  | 1.34 (0.88–2.03) |  |
| ≥15 | 1.43 (0.98–2.10) |  | 1.44 (0.98–2.10) |  | 1.43 (0.98–2.09) |  |
| Current smoker |  | 0.735 |  | 0.734 |  | 0.720 |
| No |  |  |  |  |  |  |
| Number of lost teeth |  |  |  |  |  |  |
| 0 | 1 (reference) |  | 1 (reference) |  | 1 (reference) |  |
| 1-7 | 0.95 (0.82–1.11) |  | 0.95 (0.81–1.10) |  | 0.95 (0.81–1.10) |  |
| 8-14 | 1.41 (1.03–1.94) |  | 1.40 (1.02–1.93) |  | 1.39 (1.01–1.91) |  |
| ≥15 | 1.57 (1.16–2.12) |  | 1.56 (1.15–2.10) |  | 1.54 (1.14–2.09) |  |
| Yes |  |  |  |  |  |  |
| Number of lost teeth |  |  |  |  |  |  |
| 0 | 1 (reference) |  | 1 (reference) |  | 1 (reference) |  |
| 1-7 | 1.13 (0.83–1.54) |  | 1.12 (0.82–1.53) |  | 1.13 (0.83–1.54) |  |
| 8-14 | 1.28 (0.61–2.69) |  | 1.27 (0.61–2.66) |  | 1.26 (0.60–2.64) |  |
| ≥15 | 2.37 (0.62–3.03) |  | 2.33 (0.60–2.95) |  | 2.32 (0.60–2.92) |  |
| Alcohol consumption |  | 0.320 |  | 0.320 |  | 0.325 |
| No |  |  |  |  |  |  |
| Number of lost teeth |  |  |  |  |  |  |
| 0 | 1 (reference) |  | 1 (reference) |  | 1 (reference) |  |
| 1-7 | 1.04 (0.88–1.23) |  | 1.03 (0.87–1.22) |  | 1.03 (0.87–1.22) |  |
| 8-14 | 1.20 (0.83–1.75) |  | 1.19 (0.82–1.73) |  | 1.18 (0.81–1.71) |  |
| ≥15 | 1.50 (1.08–2.08) |  | 1.49 (1.07–2.08) |  | 1.48 (1.06–2.06) |  |
| Yes |  |  |  |  |  |  |
| Number of lost teeth |  |  |  |  |  |  |
| 0 | 1 (reference) |  | 1 (reference) |  | 1 (reference) |  |
| 1-7 | 0.90 (0.72–1.12) |  | 0.89 (0.71–1.12) |  | 0.90 (0.72–1.13) |  |
| 8-14 | 1.82 (1.15–2.89) |  | 1.83 (1.15–2.90) |  | 1.81 (1.14–2.88) |  |
| ≥15 | 2.68 (0.98–2.86) |  | 2.64 (0.96–2.80) |  | 2.63 (0.95–2.77) |  |
| Regular exercise |  | 0.713 |  | 0.706 |  | 0.705 |
| No |  |  |  |  |  |  |
| Number of lost teeth |  |  |  |  |  |  |
| 0 | 1 (reference) |  | 1 (reference) |  | 1 (reference) |  |
| 1-7 | 0.98 (0.84–1.13) |  | 0.97 (0.84–1.13) |  | 0.97 (0.84–1.13) |  |
| 8-14 | 1.30 (0.94–1.79) |  | 1.29 (0.94–1.78) |  | 1.28 (0.93–1.76) |  |
| ≥15 | 1.51 (1.12–2.05) |  | 1.50 (1.11–2.03) |  | 1.49 (1.10–2.01) |  |
| Yes |  |  |  |  |  |  |
| Number of lost teeth |  |  |  |  |  |  |
| 0 | 1 (reference) |  | 1 (reference) |  | 1 (reference) |  |
| 1-7 | 1.02 (0.70–1.48) |  | 1.01 (0.69–1.47) |  | 1.01 (0.69–1.47) |  |
| 8-14 | 2.04 (1.01–4.10) |  | 2.04 (1.01–4.11) |  | 2.02 (1.00–4.06) |  |
| ≥15 | 2.74 (0.78–3.85) |  | 2.69 (0.76-3.74) |  | 2.70 (0.76–3.76) |  |
| Diabetes mellitus |  | 0.559 |  | 0.570 |  | 0.571 |
| No |  |  |  |  |  |  |
| Number of lost teeth |  |  |  |  |  |  |
| 0 | 1 (reference) |  | 1 (reference) |  | 1 (reference) |  |
| 1-7 | 0.98 (0.84–1.13) |  | 0.97 (0.84–1.13) |  | 0.97 (0.84–1.13) |  |
| 8-14 | 1.53 (1.12–2.09) |  | 1.52 (1.11–2.07) |  | 1.50 (1.10–2.05) |  |
| ≥15 | 1.92 (1.11–2.08) |  | 1.91 (1.10–2.06) |  | 1.99 (1.09–2.04) |  |
| Yes |  |  |  |  |  |  |
| Number of lost teeth |  |  |  |  |  |  |
| 0 | 1 (reference) |  | 1 (reference) |  | 1 (reference) |  |
| 1-7 | 1.01 (0.73–1.38) |  | 1.01 (0.73–1.38) |  | 1.01 (0.73–1.38) |  |
| 8-14 | 0.81 (0.34–1.89) |  | 0.81 (0.35–1.90) |  | 0.80 (0.34–1.88) |  |
| ≥15 | 2.63 (0.84–3.15) |  | 2.62 (0.84–3.13) |  | 2.61 (0.83–3.12) |  |
| Dyslipidemia |  | 0.975 |  | 0.984 |  | 0.984 |
| No |  |  |  |  |  |  |
| Number of lost teeth |  |  |  |  |  |  |
| 0 | 1 (reference) |  | 1 (reference) |  | 1 (reference) |  |
| 1-7 | 0.99 (0.85–1.15) |  | 0.98 (0.84–1.14) |  | 0.98 (0.85–1.14) |  |
| 8-14 | 1.38 (1.00–1.90) |  | 1.37 (0.99–1.88) |  | 1.35 (0.98–1.86) |  |
| ≥15 | 2.08 (1.17–2.14) |  | 2.06 (1.15–2.11) |  | 1.99 (1.14–2.09) |  |
| Yes |  |  |  |  |  |  |
| Number of lost teeth |  |  |  |  |  |  |
| 0 | 1 (reference) |  | 1 (reference) |  | 1 (reference) |  |
| 1-7 | 0.97 (0.71–1.32) |  | 0.96 (0.70–1.31) |  | 0.96 (0.71–1.31) |  |
| 8-14 | 1.45 (0.73–2.89) |  | 1.46 (0.73–2.91) |  | 1.45 (0.73–2.90) |  |
| ≥15 | 2.31 (0.60–2.88) |  | 2.36 (0.62–2.98) |  | 2.34 (0.61–2.95) |  |

Multivariable model (1) was used to evaluate the association of each oral hygiene indicator with the development of hypertension with adjustment for age, sex, income levels, regular exercise, alcohol consumption, smoking status, body mass index (kg/m^2^), diabetes mellitus, dyslipidemia, renal disease, and history of malignancy

Multivariable model (2) was used to evaluate the association of each oral hygiene indicator with the development of hypertension with adjustment for the variables in model 1 as well as systolic blood pressure, fasting blood glucose level, aspartate aminotransferase, alanine aminotransferase, gamma-glutamyl transferase, and proteinuria

Multivariable model (3) was used to evaluate the association of each oral hygiene indicator with the development of hypertension with adjustment for the variables in model 2 as well as overall oral hygiene indicators (presence of periodontal disease, frequency of tooth brushing, dental visits for any reason, professional scaling, and number of lost teeth)

CI, confidence interval; HR, hazard ratio
