# Supplementary material for: Tooth loss is associated with an increased risk of hypertension: A nationwide population-based cohort study
Source: PLoS One. 2021 Jun 15;16(6):e0253257. doi: 10.1371/journal.pone.0253257 (PMC8205122; doi:10.1371/journal.pone.0253257)
Supplement: S5 Table — (DOCX) [file pone.0253257.s009.docx]

**S5 Table. Summary of the Number of Event and Censored in this study**

| **Summary of the Number of Event and Censored** | | | |
| --- | --- | --- | --- |
| **Values** | | | |
| **Total** | **Event** | **Censored** | **Percent**  **Censored** |
| 19680 | 1853 | 17827 | 90.58 |
